# An Innovative Model of Reliability—The Pseudo-Entropic Model

**DOI:** 10.3390/e21090846

**Published:** 2019-08-30

**Authors:** Irina-Maria Dragan, Alexandru Isaic-Maniu

**Affiliations:** 1 Department of Statistics and Econometrics, The Bucharest University of Economic Studies, 010374 Bucharest, Romania; 2 Centre of Industry and Services Economy, National Institute of Economic Research, The Romanian Academy, 050711 Bucharest, Romania

**Keywords:** failure rate, entropy-like transformation, Riccati equation, reliability function

## Abstract

There is an entire field of literature on reliability models. Building reliability models is most frequently done by starting from the failure rate (or hazard rate). Creating a reliability model starts with the specific type of product behavior over time, so there are model classes for the specifics of different product categories: electronic, mechanical products, mixed systems etc. The aim of this paper was to develop a statistical model which would allow the study of the durability of products, and particularly, in the present case, of electrical switches. The procedure has a broad range of applicability and can be extended to whole categories of products that have components both in motion, and therefore subject to wear, and also that bear additional stress. In our case, an electrical switch involves the shock of the electrical contact, which additionally requires the constituent material. This article started from an indicator similar to entropy (an entropy-like transformation) that was developed according to the stated purpose of the paper. We believe that the appropriate name for the proposed indicator is pseudo-entropic transformation, as we operated with the derivative of *g*(*t*), which is basically a probability density. The model developed herein is original and, from a practical point of view, it is convenient for treating and developing analytical and practical applications for classes of products subjected, during use, to a process of wear, degradation, and damage by use.

## 1. Introduction

Reliability theory and practice uses a wide range of probabilistic models designed to respond to the great diversity of practical situations [1,2]. The most common in the profile literature are operating time to failure or between two failures (on restoring systems), represented by the frequency function or the probability density of failures.

The usual way to find different reliability functions, R(t)=Prob{T>t}, is to use the relationship:

(1) $$R\left(t\right)=\exp\left\{-\int_{0}^{t}z\left(u\right)\mathrm{du}\right\}$$

*u* ≥ 0; *z*(*u*) ≥ 0, where *z*(*u*) represents the hazard (or failure) rate associated with the *t* variable representing the time to failure of the given entity.

The associated reliability function can be formally obtained by choosing a random positive function *z*(*u*). Obviously, it is preferable that the primitive of *z*(*u*) be obtained by associated reliability function.

By choosing a certain expression for *z*(*u*), for example z(u)=const=θ>0, the exponential model f(t,a)=θexp(−at),t≥0,θ>0, can be obtained, an adequate model to describe the behavior of electronic products, which, having survived the “infant mortality” phase, enter a period of good functioning, where failures occur accidentally and rarely. Obviously, in constructing a reliability function, other functions are also suitable for the different typologies of description of the failure process or durability. The durability of products has become a major challenge in modern economies; as the complexity of products has grown, production systems have typically mixed natural, electronic, and mechanical features. Statistical techniques in industry, in an age of intense robotics and automation, have been made to face new problems, implying the need to find adequate solutions, adapted to a variety of constantly expanding situations. Statisticians’ concerns on this subject were first raised by Epstein and Sobel [3]. Gradually, this field became autonomous and today, it is considered, at least for mixed systems (mechanical-electronic), more comprehensive than reliability theory.

## 2. Literature Review

Measuring the reliability of a product can be done through a variety of methods. More than a century ago, American physicist Willard Gibbs made a famous remark, “The whole is simpler than the sum of its parts”, which has been adopted almost as an axiom by most modern engineers and economists. Gibbs [4,5] was a visionary: the vertiginous development of technology and industrial production has demonstrated that a simple component assembled on a space vehicle can itself be a true system. Runtime model to failure, whether it is the first or last, such as electric bulbs, batteries, and so on, whether it is repairable systems or components (with recovery), is, from a mathematical point of view, represented by the so-called frequency function or failure probability density function [6,7]:

(2) $$\left\{T|f\left(t;\theta \right)\geq 0,\;x\geq 0,\;\;\;\;\theta =\left(\theta_{1},\theta_{2},\ldots \theta_{m}\right),{\;\;\;\;\theta }_{j}\epsilon R,\;\;\;\;\int_{D}f\left(t;\theta \right)dt=1\right\}$$

where D=[0,+∞) is the domain of definition of the *T* variable, which denotes the service life of the product, *θ* is the parameter vector that individualizes the function *f*, and *t* represents the time.

The increasing complexity of systems makes their failure mechanisms closely linked to the states and execution quality of the system components. Analysis of failure mechanisms has been performed on abstract models of real systems [8,9,10]. One of the features of the current period is represented by a real burst of product complexity, and, as a consequence, the importance of reliability studies increases throughout a product’s lifecycle, from design to after-sales support [7]. This highlights the fact that reliability (modeling, prediction, and optimization of its level) presents a remarkably broad framework for analyzing the technical and commercial aspects of product reliability, assimilating concepts and methodologies from such diverse fields as engineering, material science, statistics, probabilistic modeling, and management. Uncertainty has a significant role in many fields, and Shannon entropy has basically resolved the uncertainty of probability theory. When uncertainty is represented by basic probability assignment (BPA) instead of probability distribution, a new entropy, named Deng entropy, has been proposed [11,12]. The proposed Deng entropy is the generalization of Shannon entropy. Pan and Deng [13] used different methods to measure uncertainty and proposed a new belief entropy without the conversion from basic probability assignment to probability, based on probability intervals and the cardinality of multiple elements of BPA. The new belief entropy has more uncertainty than other entropies, and the boundary and additivity have been improved. In mechanical engineering, some systems are very complex and might have many components, so it is likely that something might happen unexpectedly and cause serious problems due to a variety of reasons, for example having been working for a long time. In order to make a rational decision when using sensor data fusion technology, some works have been proposed to handle uncertainty. Recently, Dong et al. [14] applied belief entropy to sensor data fusion. Deng entropy was applied to measure information volume. The more information a sensor report contains, the lower the possibility that it will conflict with others. Hence, Deng entropy can be used to increase the effect of this kind of sensor report on the final decision. The new method takes into consideration not only conflict degree, but also the information volume of sensors’ outputs. Paranjape, Rajarshi, and Gore [15] introduced an interesting version for describing and measuring reliability, based on observational data and the estimation of a survival function through a graphical method, as well as an equivalent procedure, based on a linear regression, for parameter estimation and illustration of case studies. Another interesting application was developed by Li et al. [16] in the form of a probabilistic model of the reliability of an energy system and its wind energy component. Lisnianski [17] developed a relatively new issue in reliability literature, going beyond the binary test state and approaching systems with multistage reliability. His paper also accomplished a synthesis of acquisitions in multistage systems reliability research, and devoted a distinct section to the universal generating function method. Raqab and Kundu [18] considered the two-parameter Burr–Type X distribution, which can be used quite effectively in analyzing lifetime data. They also analyzed the interesting relationships with Gama, Weibull, and exponential distributions, with developments regarding the inference over the parameters of this distribution. The generation of a reliability function and all adjacent issues was developed by Wilf [19].

Lisnianski et al. [20] developed a technique to evaluate the reliability of complex systems with varying degrees of reliability, using Markov chains in a version that used a simplified method for generating the reliability function proposed by Ushakov [21], from which further reliability parameters of a system can be deduced. Li et al. also proposed a reliability calculation algorithm [22], approaching the problem of generating a reliability function. A series of investigations were developed by Vitanov [23] on generalized Riccati differential equations. In the same area of reliability function generation, Agrachev and Lee [24] made a generalization of the Ricci curve in three-dimensional space. Freiling et al. [25] investigated generalized Riccati differential and difference equations obtained from standard Riccati equations by adding a semidefinite perturbation term. For these equations, results are given on the monotonic dependence of the solutions on the coefficients and initial values, as well as results on the convergence of solutions. Oprean and Bucur [26] considered the quality of a product through an entropic approach, appreciating the loss of quality over the time of use similar to an entropic process.

## 3. The Process of Generating a Reliability Function

It is important to note that both the probability density function *F*(*t*) and the reliability function *R*(*t*) refer to events that occurred (or not) in the time period elapsed between the beginning of the operational status of the product (*t* = 0) up to the time *t* [27]. A false idea is implied by simplified form notations, which should actually be written *F*(*0*,*t*) respective to *R*(*0*,*t*), so that for a certain time, the probability of failure is written as follows:

(3) F(t,t+x)=Prob{t≤T<t+x}=F(t+x)−F(t)

where *T* represents the continuous random variable that defines the duration of operation to failure.

If the product is irreparable, then the failure is unique, and the object may fail in the range (*t*, *t* + *x*) only if it did not fail during the period (*0*,*t*). Thus, both *F*(*t*,*t* + *x*) and *R*(*t*,*t* + *x*) are probabilities conditioned by the proper functioning of the object in the range (*0*,*t*). Based on the conditional probability formula, it follows:

(4) $$F\left(t,t+x\right)=\frac{Prob\left\{t\leq T<t+x\right\}}{Prob\left\{T\geq t\right\}}=\frac{F\left(t+x\right)-F\left(t\right)}{R\left(t\right)}$$

Respectively,

(5) $$R\left(t,t+x\right)=\frac{Prob\left\{T\geq t+x\right\}}{Prob\left\{T\geq t\right\}}=\frac{R\left(t+x\right)}{R\left(t\right)}$$

These functions describe the behavior of a product over a given timeframe; thus, for a precisely given moment, we use the probability density:

(6) $$f\left(t\right)=\underset{\Delta t\to 0}{\lim}\frac{F\left(t+\Delta t\right)-F\left(t\right)}{\Delta t}=\frac{dF\left(t\right)}{dt}$$

which is actually the limit of the ratio between the total probability of failure in the range (t,t+Δt) and the length (Δt) when this length tends to zero.

The fundamental indicator is the "hazard rate" or "failure rate", which describes the so-called “danger of failure” at a given moment of a technical or biological system knowing that it was operational up to that point.

Yet again, the conditional probability formula gives:

(7) $$z\left(t\right)=\underset{\Delta t\to 0}{\lim}\frac{F\left(t+\Delta t\right)-F\left(t\right)}{R\left(t\right)\Delta t}=\frac{f\left(t\right)}{R\left(t\right)}$$

which can also be written as

(8) $$z\left(t\right)=-\frac{1}{R\left(t\right)}=\frac{dR\left(t\right)}{dt}$$

which represents the simple differential equation with the condition at the limit R(0)=1.

This immediately results in

(9) z(t)dt=−d[lnR(t)]

from which, through integration, can be obtained

(10) $$lnR\left(t\right)=-\int_{0}^{t}z\left(u\right)du$$

which is:

(11) $$R\left(t\right)=exp\left\{-\int_{0}^{t}z\left(u\right)du\right\}$$

the relationship that links the failure rate to the other indicators.

Further, we can derive Equation (11) and immediately obtain:

(12) $$f\left(t\right)=z\left(t\right)\cdot exp\left\{-\int_{0}^{t}z\left(u\right)du\right\}$$

which is one of the fundamental relationships from which various statistical reliability models can be generated.

It is important that from Equation (12), the density *f*(*t*) can clearly express itself; that is, the primitive of *z*(*u*) is calculated by elementary functions, which obviously facilitates the mathematical approach. The option for *z*(*u*) = θ > 0 provides the exponential model [27,28]:

(13) f(x:θ)=θ·exp{−θx},    x≥0,    θ>0

Equation (12) can also be deduced, for reasons that are not bound to the initial reliability significance of the *f* and *z* functions. Let *f* and *z* be two continuous, strictly positive functions for x∈(0,+∞), that satisfy the equation:

(14) $$\frac{df}{f}=\frac{dz}{z}-z$$

with conditions $\underset{\begin{array}{c} x\to 0\\ x>0 \end{array}}{\lim}\frac{f\left(x\right)}{z\left(x\right)}=1$ and $\underset{x\to \infty }{\lim}\frac{f\left(x\right)}{z\left(x\right)}=0$, in which case *f* is as follows:

(15) $$f\left(x\right)=z\left(x\right)\cdot exp\left\{-\int_{0}^{x}z\left(t\right)dt\right\}$$

which gives $\int_{0}^{\infty }f\left(x\right)dx=1$, so *f*(*x*) is a probability density.

Equation (15) results from writing Equation (14) as d(lnf)=d(lnz)−z, which, integrated on the interval $\left(x_{0},x\right),0<x_{0}<x$, leads to $\ln f\left(x\right)-\ln f\left(x_{0}\right)=\ln z\left(x\right)-\ln z\left(x_{0}\right)-\int_{x_{0}}^{x}z\left(t\right)dt$, or which is identical to $\ln f\left(x\right)-\ln z\left(x\right)=-\int_{x_{0}}^{x}z\left(t\right)dt+\ln\frac{f\left(x_{0}\right)}{z\left(x_{0}\right)}$.

When $x_{0}\to 0$, and considering that $\underset{x\to 0}{\lim}\ln\frac{f\left(x_{0}\right)}{z\left(x_{0}\right)}=\ln1=0$:

(16) $$f\left(x\right)=exp\left\{\ln z\left(x\right)-\int_{0}^{x}z\left(t\right)dt\right\}=z\left(x\right)\cdot exp\left\{-\int_{0}^{x}z\left(t\right)dt\right\}$$

which is Equation (15). It should be pointed out and proven that f is indeed a probability density. Equation (14) also can be written as $\frac{zdf-fdz}{z^{2}}=-f$ or $d\left(\frac{f}{z}\right)=-f$ from which, on the same interval $\left(x_{0},x\right)$, can be obtained:

(17) $$\int_{x_{0}}^{x}d\left(\frac{f}{z}\right)dt=-\int_{x_{0}}^{x}f\left(t\right)dt$$

Thus

(18) $$\frac{f\left(x\right)}{z\left(x\right)}-\frac{f\left(x_{0}\right)}{z\left(x_{0}\right)}=-\int_{x_{0}}^{x}f\left(t\right)dt$$

Developing to the limit, for $x_{0}\to 0$ we get:

(19) $$\frac{f\left(x\right)}{z\left(x\right)}-1=-\int_{0}^{x}f\left(t\right)dt$$

which for x→∞ results $\int_{0}^{\infty }f\left(t\right)dt=1$.

Obviously, it can be concluded from Equation (19) that:

(20) $$z\left(x\right)=\frac{f\left(x\right)}{1-\int_{0}^{x}f\left(t\right)dt}$$

and having previously argued that *f* is a density, it is obvious that, in this case, z(x) it is exactly the failure intensity.

This equation results from the Riccati differential equation [23,24,29]:

(21) $$y^{\prime }=P\left(x\right)y^{2}+Q\left(x\right)y+R\left(x\right)$$

where R≢0,P,Q,R being continuous functions on the (0,+∞) interval.

Making the following choices $P\left(x\right)\equiv 0,\;Q\left(x\right)=\frac{f^{\prime }\left(x\right)}{f\left(x\right)},\;R\left(x\right)\equiv -1,$ where *f* is a strictly positive and continuous function (0, +∞), the Riccati equation becomes: $y^{\prime }=-\frac{f^{\prime }}{f}\cdot y-1$ or $f\cdot y^{\prime }+f^{\prime }\cdot y=-f.{\left(f\cdot y\right)}^{\prime }=-f$.

By integrating this equation on $\left(x,x_{0}\right),\;0<x<x_{0}$ results in:

(22) $$f\left(x_{0}\right)\cdot y\left(x_{0}\right)-f\left(x\right)\cdot y\left(x\right)=-\int_{x}^{x_{0}}f\left(t\right)dt$$

considering the transformation y(x)=1/z(x), where z(x) is a strictly positive and continuous function on (0,+∞) interval. In a transformation of this type, the Riccati equation preserves its shape and results: $\frac{f\left(x_{0}\right)}{z\left(x_{0}\right)}-\frac{f\left(x\right)}{z\left(x\right)}=-\int_{x}^{x_{0}}f\left(t\right)dt$, therefore

(23) $$z\left(x\right)=\frac{f\left(x\right)}{\frac{f\left(x_{0}\right)}{z\left(x_{0}\right)}-\int_{x}^{x_{0}}f\left(t\right)dt}$$

If there is the condition $\underset{x_{0}\to \infty }{\lim}\;\frac{f\left(x_{0}\right)}{z\left(x_{0}\right)}=0$, then

(24) $$z\left(x\right)=\frac{f\left(x\right)}{\int_{x}^{\infty }f\left(t\right)dt}$$

(25) $$\frac{z^{\prime }\left(x\right)}{z\left(x\right)}=\frac{f^{\prime }\left(x\right)}{f\left(x\right)}+\frac{f\left(x\right)}{1-F\left(x\right)}\;\;\;\;\;\;\;\;\;\;\;\;\;\left(F^{\prime }\left(x\right)=f\left(x\right)\right)$$

is useful for the study of failure intensity behavior, or more precisely for locating its maximum (if any)—an important issue for technique applications.

From Equation (25), it can be remarked that if $f^{\prime }\left(x\right)\geq 0$, then $z^{\prime }\left(x\right)>0$, but if $f^{\prime }\left(x\right)<0$, then $z^{\prime }\left(x\right)$ can take any value, positive, null, or even negative. Let us notice that:

(26) $$z^{\prime }\left(x\right)={\left[\frac{f\left(x\right)}{1-F\left(x\right)}\right]}^{\prime }=\;\frac{f{\left(x\right)}^{\prime }\cdot \left[1-F\left(x\right)\right]+f^{2}\left(x\right)}{{\left[1-F\left(x\right)\right]}^{2}}$$

and therefore, the behavior of z(x) can be studied directly from Equation (26) if the distribution function is explicit.

## 4. The Pseudo-Entropic Model of Reliability

Soleha and Sewilam [30] considered the random variable *T* representing the proper functioning time of any component and introduced the following expression:

(27) g(t)=F(t)+R(t)lnR(t)

where F(t) and R(t)=1−F(t) are, respectively, the distribution and the reliability function of a positive continuous random variable *T*.

They named this form of g(t) an “entropy-like transformation”, probably due to the term R(t)lnR(t), which resembles the expression of entropy associated with a continuous variable *X* with the density f(x):

(28) H=−∫f(x)lnf(x)dx

We prefer to use for Equation (27) the term “pseudo-entropy transformation”, because, in fact, we work with the derivative of g(t), that is:

(29) $$g^{\prime }\left(t\right)=F^{\prime }\left(t\right)+R^{\prime }\left(t\right)lnR\left(t\right)+R\left(t\right)\cdot \frac{R^{\prime }\left(t\right)}{R\left(t\right)}=f\left(t\right)+R^{\prime }\left(t\right)lnR\left(t\right)-f\left(t\right)=R^{\prime }\left(t\right)lnR\left(t\right)$$

because $F^{\prime }\left(t\right)=f\left(t\right)$ and $R^{\prime }\left(t\right)={\left[1-F\left(t\right)\right]}^{\prime }=-F^{\prime }\left(t\right)=-f\left(t\right)$.

The form $g^{\prime }\left(t\right)=R^{\prime }\left(t\right)lnR\left(t\right)$ is basically a probability density, and thus can be used in describing the reliability as it fulfills the condition:

(30) $$\int_{0}^{\infty }g^{\prime }\left(t\right)dt=\int_{0}^{\infty }R^{\prime }\left(t\right)lnR\left(t\right)dt=1$$

Additionally,

(31) $$G\left(t\right)=1-\int_{0}^{t}R^{\prime }\left(x\right)lnR\left(x\right)dx$$

is the reliability function associated with the *T* variable.

Obviously, Equation (31) is not an entropy, since the derivative of R and not R appears under the integral—as in the general Equation (28)—hence, the name proposed for the present process is pseudo-entropy.

Thus, we worked with the analytic Equation (29) as a probability density, and we observed the following: the model $u\left(t\right)=R^{\prime }\left(t\right)lnR\left(t\right),\;t\geq 0$ is suitable from the analytical approach point of view, if the function of reliability R(t), the generator of the model itself, is of exponential type, that is: R(t)=exp[−θ(t)], where θ(t)≥0 for every t≥0 and, in this case, u(t) becomes:

(32) $$u\left(t\right)=\theta \left(t\right)\theta^{\prime }\left(t\right)\cdot exp\left[-\theta \left(t\right)\right]$$

Thus, if we consider $\theta \left(t\right)=at^{2},\;a>0,\;t\geq 0$ we will obtain the frequency function

(33) $$u\left(t\right)=2a^{3}t^{3}exp\left(-at^{2}\right),\;t\geq 0,\;a>0$$

which is just a particular case of the generalized Rayleigh model [31].

If we propose for θ(t) the linear form θ(t)=at, a>0, t≥0, it results

(34) $$u\left(t\right)=a^{2}t\;exp\left(-at\right),\;t\geq 0,\;a>0$$

which is the reliability function associated with the form (1+at)exp(−at), and the failure rate is of homographic type, namely

(35) $$h\left(t;a\right)=\frac{a^{2}t}{1+at}=a\left[1-\frac{1}{1+at}\right]$$

therefore, this is seen to be an increasing function on the interval [0,∞) so it can be associated with the phenomenon of wear or degradation over time of material properties, loss of quality through use, and so on.

The main indicators of the *T* variable are: mean E(T)=2/a, variance $Var\left(T\right)=2/a^{2}$, coefficient of variation $CV\left(T\right)=1/\sqrt{2}$, asymmetry coefficient (usually called the coefficient of skewness) $\beta_{1}=\sqrt{2}$, and coefficient of excess (usually called the coefficient of kurtosis) $\beta_{2}=6$. Hence, it can be concluded that the distribution described by Equation (34) is asymmetrical to the right, having a more acute peak around the mean and fatter tails than the Gauss-Laplace distribution. It is useful to notice an interesting property: $\beta_{1}=2\cdot CV\left(T\right)$, meaning that the asymmetry coefficient, as a value, is double the coefficient of variation.

## 5. Testing the Model and Conclusions

An ordinary criticism for theoretical researchers in quality science is that, in some cases, the models proposed for the behaviors of different product quality features do not instantly or always correspond to the real world [32,33,34,35], but this is a way of reducing and simplifying the interaction between theory and practice. Frequently, the theory outstrips the practical possibilities of its illustration and application.

A statistical model, like the one presented here, has theoretical justification and can illustrate a real case with collected data. For such an example, a sample of 100 electrical switches were randomly extracted and connected to a test bench for electronic devices. These appliances were subjected to a durability test that consisted of 40 operations (closed-open cycles) per minute. Experimental data were recorded at the company Roman S. A. in Brasov [36] in October–November 2017. Roman S.A. is a truck and bus manufacturer from Brasov, Romania. It also manufactures various components for trucks like engines, axles, decks etc. Obtaining the necessary information is accurate because, in the case of complex processing systems, the maintenance division records the malfunctions, therefore, the distribution of failures can be made at certain intervals with relative ease, and then a reliability model can be established, including the one proposed by us, the pseudo-entropic model. Moreover, periodically, by repeating the operations, a description of the system behavior over time can be made. This information (Table 1), are presented below as $t_{i}\left(n_{i}\right)$ where $t_{i}$—operations, $n_{i}$—absolute frequencies.

Thus, the mean value was 8.73 and the standard deviation was 6.176. Therefore, the coefficient of variation was 0.707 and, accordingly, $\frac{1}{\sqrt{2}}=0.707098$, so the model was appropriate for the experimental data considered.

The results obtained for sample data validated the proposed reliability model, which is an alternative to compiling models describing the reliability of complex systems marked by degradation or wear during normal operation.

The construction of some probability models for reliability is subordinate to the diversity and complexity of products, differentiating: with or without restoration, with or without manufacturing deficiencies, with sudden failure, not preceded by symptoms, specific for electronic products, with gradual deterioration, specific to the mechanical systems, and respectively to electronic-mechanical combinations, the option most frequently encountered in complex systems, or after the way of connecting the system components: serial, parallel, or combined, or caused by the aging or degradation of materials. Each of the failure modes had its own statistical behavior and therefore its own reliability function, which described the evolution of the indicators over time.

The proposed procedure is particularly versatile, being recommended to describe the reliability of a broad range of complex products that are subjected to a degradation process during use, their physical state being affected by wear and tear resulting from repeated uses. Furthermore, it is recommended for the measurement and characterization of the reliability for the products subjected periodically to extreme strain (e.g., on/off cycles) as is the case of mechanisms with discontinuous functioning, as well as in the cases of some tension with variable intensity. The proposed procedure is also useful for accelerated life testing, developed in order to decrease the time taken to obtain the results of reliability assessments, in fatigue-testing, and in stress strength tests. These technical methods of testing highlight the fact that the qualitative performances of the products are lost over time; thus, reliability is seen as an entropic process.

This paper presented a statistical approach for building a reliability model specific to wear-resistant components during use. The novel aspect of the process is in the relative resemblance to an entropic indicator. The sample tested application confirmed the correctness of the undertaken approach, and illustrated and completed the diversity of the means for projecting some probabilistic models for reliability computing.

## Figures and Tables

**Table 1:** Results^1^ of the test bench for electronic devices.

1 (2)	2 (1)	3 (5)	4 (13)	5 (20)	6 (8)
7 (6)	8 (9)	9 (6)	10 (7)	11 (5)	12 (1)
13 (3)	14 (3)	15 (1)	16 (3)	18 (3)	19 (2)
21 (1)	24 (1)	26 (1)	27 (1)	39 (1)	

^1^ Sample data collected at Roman S. A.
